# Community and health staff perceptions on non-communicable disease management in El Salvador’s health system: a qualitative study

**DOI:** 10.1186/s12913-020-05249-8

**Published:** 2020-05-27

**Authors:** Nicole Vidal, Montserrat León-García, Marta Jiménez, Keven Bermúdez, Pol De Vos

**Affiliations:** 1 Institute for Global Health and Development, Queen Margaret University, Edinburgh, UK; 2 Biomedical Research Institute Sant Pau (IIBSant Pau), Iberoamerican Cochrane Centre, Universidad Autónoma de Barcelona, Barcelona, Spain; 3 Institute of Tropical Medicine, Antwerp, Belgium

**Keywords:** Non-communicable diseases, Community health, El Salvador, Qualitative methods

## Abstract

**Background:**

Non-communicable Diseases (NCDs) are the leading cause of global mortality and disability with a rising burden in low- and middle-income countries. Their multifactorial aetiology, and their requirement of long-term care, implies the need for comprehensive approaches. From 2009, the Ministry of Health (MoH) in El Salvador has developed a national public health system based on comprehensive primary health care. This study aims to describe the different stakeholders’ perceptions about the management of NCDs along the pathways of care in this health system.

**Methods:**

During three fieldwork periods in 2018, three complementary qualitative data collection methods were deployed and conducted in settings with high prevalence of NCDs within El Salvador. First, illness narrative methodology was used to document the life histories of people living with a chronic disease and being treated in second and third level health facilities. Second, through social mapping, support resources that NCD patients used throughout the process of their illness within the same settings were analysed. Third, semi-structured interviews were conducted in the same locations, with both chronic patients and health personnel working at different levels of the primary health care setting. Participants were recruited through purposive and snowball sampling, and a deductive approach was implemented for coding during the analysis phase. After grouping codes into potential themes, a thematic framework was developed using a reflexive approach and following triangulation of the data.

**Results:**

This innovative approach of combining three well-defined qualitative methods identified key implications for the implementation of a comprehensive approach to NCD management in resource-poor settings. The following elements are identified: 1) social risk factors and barriers to care; 2) patient pathways to NCD care; 3) available resources identified through social connections mapping; 4) trust in social connections; and 5) community health promotion and NCD prevention management.

**Conclusions:**

The Salvadoran public health system has been able to strengthen its comprehensive approach to NCDs, combining a clinical approach – including long-term follow-up – with a preventive community-based strategy. The structural collaboration between the health system and the (self-) organised community has been essential for identifying failings, discuss tensions and work out adapted solutions.

## Background

Non-communicable diseases (NCDs) are a major health priority in low- and middle-income countries (LMICs) [1]. In 2016, over three quarters of NCD deaths (31.5 million) took place in these countries, with nearly half of these deaths occurring before the age of 70 [2]. Though largely preventable, these illnesses are the leading cause of global mortality and disability, and have complex social, economic and environmental aetiologies [3, 4]. The burden created by NCDs undermines individual and family well-being and hampers social and economic development, with a particular threat to vulnerable populations [5].

In El Salvador, NCDs are a leading cause of early death among the adult population. Lifestyle behaviours such as low consumption of fruit, vegetables and water, excessive consumption of saturated fats and sugary drinks, but also exposure to agrochemicals, are the main risk factors and are directly linked to social and structural barriers. During the period of 2011–2015, the Ministry of Health (MoH) recorded 48,554 NCD deaths, with cardiovascular diseases accounting for 12.0% of the total deaths registered nationwide. This is followed by chronic kidney disease (CKD) at 6.3%, cancer at 5.4%, and diabetes mellitus type 2 (DMT2) at 3.0% [6] Though the World Health Organisation (WHO) does not consider CKD to be part of the NCD group, it was included in this study due to its high prevalence in the country.

Since 2009 – within a context of high levels of poverty and social violence – the Salvadoran government developed a strong primary health care (PHC) policy, increasing access to care, with emphasis on remote populations [7]. In this context, the current study explores the different stakeholders’ perceptions of the management of NCDs along the pathways of care in this health system. Our hypothesis is that a well-structured and sufficiently resourced PHC system can ensure adequate pathways for diagnosis, care and long-term treatment for people living with NCDs.

Due to the high burden of NCDs, there is a growing interest in effective strategies to address complex community health issues, especially when health systems face significant resource constraints [8]. Hence, this study also describes the extent to which an intensive coalition between the public health care system and an organised community can support a comprehensive approach to NCD care. For this, three complementary qualitative approaches were combined and conducted at different levels of the health system. At the hospital level, we elicited narratives of patients with a long NCD history on their illness experiences and health seeking. In communities, we mapped health care resources reported by patients and how these were used. Within primary health services, we examined the experiences of both patients and PHC providers in the diagnosis and management of NCDs. This study is in line with other studies reporting on resource-scarce countries. During a trial in Cuba for instance, Stein and Susser [9] had the opportunity to observe the Cuban health system whilst undertaking a consultancy for the WHO. They found that post-revolutionary Cuba had placed a high priority on health, and aimed to create a health service that is comprehensive in scope and content, in population covered, and in organisational forms and levels of specialisation. They highlight that their achievements cannot be understood without an appreciation of the social and political context within which they occurred. Furthermore, community-based NCD interventions have been shown to be effective in the management of NCDs in developing countries [10]. Their study reveals how programmes should be planned, run and evaluated according to clear principles and rules, and how they should collaborate with all sectors of the community while maintaining close contact with the national authorities [10]. This is a novel study as it describes how NCDs are managed in a low-resource setting by taking into account not only a comprehensive health system approach but also the social, economic and political environment that impact on NCD care, thereby including the communities in their own healthcare management.

El Salvador, the smallest country in Central America, has suffered several periods of armed conflict during its history. The twentieth century alone included a military dictatorship from the early 1930s to 1979, and a civil war from 1980 to 1992, during which 75,000 civilians were killed. A long and complex process led to a peace agreement in 1992 and the transition to formal democracy. In this period, the Farabundo Marti National Liberation Front (FMLN), a leftist guerrilla organisation, became a political party, which later played a key role in the development of a comprehensive health reform. From 1992 until 2008, neoliberal governments ruled the country. This period saw structural neglect of the public health system accompanying health sector privatisation, further developing an already dramatically socially depressed country [11].

Currently, the war between gangs (“*maras”*) among the young population remains a serious social problem, leading to displacements and thousands of deaths every year. The United Nations (UN) has classified El Salvador as one of the deadliest countries in the world outside of a war zone, with more than 108 homicides per 100,000 inhabitants in 2015. Young people are the most affected by violence: more than half of the victims of homicide in El Salvador, Guatemala and Honduras in 2015 were under the age of 30. El Salvador continues to have one of the highest murder rates in the world with 3605 homicides per year in 2017; 429 of which were femicides [12, 13]. From 2009 until 2018, the FMLN played an important role in the government. The *right to health* was core to their political programme. The new MoH embraced and further developed community participation in the preparation, development and management of the health reform [14, 15].

Over the last decade, El Salvador has developed a strong integrated public health system, in which organised social participation – defined and implemented as a structured democratic space for the accountability of health care institutions – played an essential role in building trust between the population and the public health system [14]. The Salvadorian approach builds on the extensive experience of the People’s Health Movement and shows how organised communities can support – or even accelerate – the implementation of comprehensive approaches to community health. The struggle for accessible health care has always been part of a broader movement for health equity and social change [16]. Moreover, health providers play a strong supporting role in strengthening people’s empowerment [17]. Opportunities for this depend on the context of national health policies and existing levels of community organisation or involvement. In El Salvador, the National Health Forum (NHF), a broad national movement of organised civil society, strongly supported the rollout of the health reform, encouraging community participation at all levels.

Through its empowering strategies and active mobilisation, the NHF aimed to strengthen the participation of community leaders in the co-management and control over health policies, practices and services. The movement also mobilised in support of the health reform’s efforts towards universal access, universal health coverage, and the pursuit of equity [18].

Within this context, the MoH launched its “*Multi-sectoral National Strategic Plan towards the Comprehensive Management of Non-Communicable Diseases in El Salvador*” [19]. The plan implemented interventions for the prevention and control of NCDs recommended by the WHO Global Action Plan of NCDs 2013–2020 [20].

It addressed three aims: first, the development of programmes for NCD management, aiming at prompt prevention and effective treatment, pursuing an efficient system of early diagnosis, and the development of educational programmes; second, the coordination of activities with other public institutions and with community organisations for the prevention and control of NCDs, aiming at a comprehensive and multidisciplinary national programme; and third, multi-sectoral interventions with broad social participation to reduce NCD morbidity and mortality. Hence, our study also describes the role that social participation played in the implementation of these proposals, and the lessons for structured processes of social engagement with care and prevention of NCDs in settings of fragility.

The overall rationale of using these three well-defined qualitative methods at the hospital, primary health care and community levels was to obtain an in-depth understanding of NCD management in El Salvador, along the pathway of care in order to identify key implications for the implementation of a comprehensive approach to NCDs in resource-poor settings.

This study relates to other global health research conducted under the auspices of the NIHR Research Unit on Health in Fragility (RUHF), at the Institute for Global Health and Development at Queen Margaret University (QMU), emphasising strengthening the provision of quality care for NCDs and mental health in fragile settings including Syrian refugees and host communities in Lebanon and fragile communities in Sierra Leone, and El Salvador.

## Methodology

### Study setting

This study was conducted in two urban and five rural locations across five departments of El Salvador: the departments of San Salvador and San Miguel (phases 1 and 2), Usulután (phase 2), and Chalatenango, Morazán and Bajo Lempa, which is also located in Usulután (phase 3). The health facilities visited, which were located in the central, oriental and metropolitan regions, were selected to represent variation in size of health infrastructure, population served, geographical characteristics, and prevalence of chronic conditions (Table 1). Access to data collection sites was facilitated by members of the MoH, social movement members, and medical staff from the health facilities where data were collected.

**Table 1:** Prevalence of chronic conditions in El Salvador

	Diabetes mellitus (DM)	Hypertension (HBP)	Chronic kidney disease (CKD)
NATIONAL LEVEL	487,875	1,446,381	459,114
REGION			
Occidental (Ahuachapán, Santa Ana, Sonsonate)	86,524	296,275	74,181
Central (**Chalatenango**^a^, La Libertad)	52,274	141,189	23,634
Paracentral (Cuscatlán, La Paz, Cabañas, San Vicente)	53,799	180,519	82,133
Oriental (**Usulután**^a^, **San Miguel**^a^, **Morazán**^a^, La Unión)	101,084	297,210	150,990
Metropolitana (**San Salvador**^a^)	194,194	531,187	128,176
SEX			
Male	179,708	604,184	283,905
Female	308,167	842,196	175,210

^a^Regions where health facilities are located

### Study design

Along with document review, this study used three complementary qualitative methods over three stages of data collection to give voice to participants and gain an in-depth understanding of patient and provider experiences related to NCD care:

1. Illness narratives of patients with chronic kidney disease (CKD) and Diabetes Mellitus type 2 (DMT2) developed through in-depth interviews with patients in both national and regional public hospitals. The aim of using this method was to gain understanding of the social risk factors, barriers to care and patient pathways to NCD care.
2. Mapping social connections of NCD patients with the aim to obtain an insight into patients’ social networks. For this, an adapted version of a participatory tool [21] was used. The original tool, designed to map social relationships in humanitarian contexts to gain an understanding of the social resources available to communities affected by conflict [21], was modified for this study to provide detail on the social networks of NCD patients in the specific fragile conflict-affected context of El Salvador. This method equally enables communities to participate in identifying levels of connection and trust among the people and organisations who provide support with their NCD management. The tool was implemented with the technical support of QMU researchers who have applied its use in other fragile and conflict-affected settings.
3. Semi-structured interviews with patients and staff members in the PHC setting aimed at understanding the pathways of NCD care within the health system from the viewpoint or perception of the participants, emphasising community perspectives and the role of social participation to strengthen NCD services. This method was aimed at gaining understanding of community health promotion, prevention and management of NCDs as well as the role of the NHF in providing NCD care.

### Data collection

Data were collected to investigate the resources available for managing NCDs at two levels of care: specialised and primary health care (including at the community level). Fieldwork took place over three respective phases, in November 2017, March 2018 and June 2018.

#### Patient illness narratives using in-depth interviews

Illness narratives are a useful means to access patients’ perspectives on illness. They provide a way to gain an in-depth understanding of the pathways of care and treatment of people living with NCDs, taking the sociocultural and political context into account [22]. The research team decided to perform the illness narratives over the first phase of data collection as they raised questions and problems related to NCD management, which could be further investigated at the community and primary health care level.

Through in-depth interviews, six illness narratives were collected during the first phase of data collection. Participants were recruited through purposive and snowball sampling in two separate Salvadoran hospitals which have specific epidemiological and geographical characteristics in regard to NCDs. Participants included hospitalised patients who were identified and recruited by health care staff unaware of the research objectives and who met the following inclusion criteria: (1) being aged 35 or older, (2) having been diagnosed with DMT2 or CKD, and (3) exhibiting lucidity and thereby the ability to freely consent to participate in this study. The age group was determined based on the WHO-defined “higher risk group” for NCDs [23]. Patients suffering from CKD and DMT2 were purposively selected for illness narrative interviews considering the high prevalence of these diseases in the country (487,785 patients with CKD and 459,114 with DMT2) [24] and also because of the social factors involved in the aetiology of both diseases (e.g. diet, access to fresh water, sedentarism, violence, pesticide use).

Firstly, three of the illness narratives were obtained with patients at Rosales Hospital, the national public hospital of El Salvador, located in the metropolitan region where the majority of NCD cases are managed [6]. Patients were selected from this hospital as it attends to people from all over the country. As such, the research team was able to explore the different experiences and pathways of support among patients coming not just from the capital but also from very remote areas of El Salvador. Moreover, it is the main hospital where severe complications of NCDs are treated.

Secondly, three interviews were conducted at San Juan de Dios Hospital, in the city of San Miguel, located in the eastern part of the country (oriental region), where there is the highest incidence of CKD. This hospital is a referral public hospital for CKD management with an interdisciplinary approach and promotion of ambulatory peritoneal dialysis. The hospital also has a dialysis unit, which is where interviews were conducted. Patients were selected from this hospital given its rural location as well as to explore CKD management in detail as it is a less commonly named NCD yet highly prevalent in the country, with El Salvador rating among the highest worldwide [25].

The interviews elicited information on the patients’ background, family history, social networks and health, with emphasis on detailed patient experiences of treatment initiation, follow-up, care and support, communication with providers at each stage of treatment, and the broader social and structural determinants of health care accessibility and quality.

The illness narratives were developed in the hospital environment including the endocrinology unit, nephrology unit and dialysis unit. As hospital settings are usually the last step of care, they provide an opportunity to explore with participants the “causes of the causes” and the different stages in the development of the disease and pathways of care [23]. Furthermore, information on existing support through social networks and community structures was also included in the illness narrative interviews.

#### Mapping participants’ social connections using a social connections mapping tool

The second phase of this study aimed at gaining insight into the formal and informal support resources available to people with NCDs by mapping their network of social connections and their levels of trust with the connections identified.

Mapping participants’ network of connections was ensured through a series of participatory mapping workshops, designed to elicit awareness of local social connections and resources. Participants were recruited through purposive and snowball sampling facilitated by NHF and health care staff. Inclusion criteria for participants included: (1) being aged 35 or older, (2) having an NCD diagnosis, (3) being affiliated to the NHF (for participants belonging to the “organised” communities), and (4) exhibiting lucidity and thereby the ability to freely consent to participate in this study.

**Fig. 1:**
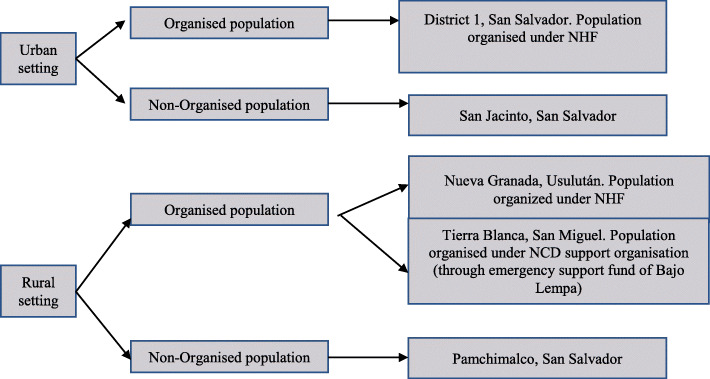
Communities included in social mapping exercise

Participants were asked about the different people and organisations in their communities whom they identified to be helpful if encountering problems during the stages of care. Five workshops of four participants each were carried out to cover communities organised under the auspices of the NHF social movement, as well as those that were not organised under the NHF movement. Workshops were held in two urban areas of San Salvador: District 1 (organised group in an urban setting) and San Jacinto (non-organised group in an urban setting), and three rural areas: Nueva Granada, Usulután (organised group in a rural setting); Tierra Blanca, San Miguel (organised group in a rural setting); and Pamchimalco, San Salvador (non-organised group in a rural setting) (Fig. 1). These locations were selected to capture urban and rural settings as well as to be near the hospitals assessed in phase 1 where more cases of NCDs are present.

During the workshops, participants were presented with three disease-based problem scenarios – diagnosis, acute episodes, and follow-up – in order to determine whom they might contact and to whom each of these connections might pass them on to, if necessary. Responses were plotted on paper linking persons or organisations to the problems for which they were accessed.

After mapping social connections and resources in the different stages of care, participants were interviewed individually to determine the level of trust placed in each of the identified sources. For all connections identified, participants were asked to grade the level of trust they placed on each. This process was facilitated through a card sorting activity, consisting of the researchers producing a set of cards with each of the identified connections written clearly on each. Participants then graded the cards by placing them in one of three piles representing either no trust in that resource, some or little trust in that resource, or high trust in that resource. There was no overlap in sampling and recruitment of participants between phase 1 (illness narratives) and phase 2 (social mapping) and the participants were not informed about the other phases of the research at any time.

#### Semi-structured interviews with NCD patients and staff members in the first line setting

The third phase of data collection involved conducting semi-structured interviews with NCD patients and their health providers, building on the findings from the illness narratives and social connections mapping. The objective was to gain an extensive understanding of patient and provider experiences with NCD care in the first line setting.

The study population consisted of staff members working in PHC facilities and of patients with one or more NCD attending these units. Patient inclusion criteria included: (1) being aged 35 or older, (2) living with an NCD, and (3) receiving care from the PHC units.

Purposive and snowball sampling methods were used to recruit participants. Purposive sampling, which is useful for identifying hard to reach populations, was used to locate participants from specific communities. Staff members and patients were initially identified by referral from the health unit coordinators, who assessed eligibility. A chain referral system was then used by asking participants to recommend any other staff members or patients they were aware of who met the recruitment criteria and might be interested in participating in this study.

Interviews were conducted with 14 NCD patients and 12 staff members using a semi-structured interview guide developed specifically for this study (Additional file 1). Participants were interviewed at their place of choice, in either the workplace or domicile.

Patient interviews covered aspects of their experience seeking and/or receiving care at the PHC facility, and included questions on treatment initiation, care and support, communication with providers at each stage of treatment, and the broader familial and social context of their medicine-taking (or other aspects of care seeking) behaviour.

The 14 NCD patients interviewed suffered from cardiovascular disease, DMT2 and/or CKD (Table 2). The median age of those interviewed was 64 years old. Staff member interviews were included to incorporate a comprehensive understanding of the PHC system by presenting the providers’ perspective of NCD care. Health providers working in different levels within PHC were selected to participate (Table 3).

**Table 2:** NCD patient interviews

Non-communicable diseases suffered by patients interviewed	N
Cardiovascular disease	9
Diabetes Mellitus type 2	3
Chronic Kidney Disease	2
Total	14

**Table 3:** Health provider interviews

Primary health care staff	Professional profile	N
Coordination level	Departmental coordinator (1)	4
	Intermunicipal coordinator (2)	
	Regional coordinator (1)	
Interdisciplinary PHC team	Health educator (1)	8
	Sanitary inspector (1)	
	Medical student in year of social service (1)	
	General practitioner (1)	
	Family doctor (1)	
	Nurses (1)	
	Pharmacist (1)	
	Laboratory technician (1)	
Total		**12**

### Data management and analysis

All interviews and participatory workshops were conducted by the researchers in Spanish, and were digitally recorded and subsequently transcribed into Microsoft Word documents. Audio recordings were permanently deleted following transcription. All qualitative data were then coded, both manually and using NVivo Qualitative Analysis Software version 10.0.

Data collected from all three methods were analysed separately. A deductive approach was used to develop an open coding system around the topics of patient pathways to care, and patient and provider interactions and experiences of care. All data were then triangulated to identify consistencies or variations across the dataset. The last step involved collating the coded data into a unique pre-defined thematic framework (Table 4).

**Table 4:** Thematic framework

Illness narratives	1) Social determinants of care 2) Patient pathways 3) Health systems issues (e.g. costs of care, waiting times, quality of care)
Mapping social connections and trust allocation	1) Public institutions (e.g. primary health care centres, public hospitals, maternity health centres, social insurance services) 2) Private institutions (e.g. private clinics, private hospitals, private doctors, naturopath) 3) Organisations (including local and international NGOs) 4) Community (e.g. neighbours, community associations) 5) Family (e.g. immediate or extended family members)
Semi-structured interviews with NCD patients and staff members in the PHC setting	1) Primary health care in El Salvador (including organisation and coordination of care, evolution of the country’s health system, accessibility and barriers to care) 2) Chronic disease management in the PHC system (including prevention and promotion of care, pathways to care, quality of care and barriers to care) 3) Community engagement

### Ethical considerations

This study was approved by the National Health Ethics Committee of El Salvador (CNEIS/2018/005_A) and by the ethics committee of Queen Margaret University in Edinburgh. All participants were provided with an information sheet detailing the objectives of the study and their rights as participants. Written informed consent was obtained from each participant prior to their involvement, with participants being informed of their right to decline to take part and/or leave at any time.

## Findings

From across the three sources of data collection, the following key issues were analysed:

1) social risk factors and barriers to care; 2) patient pathways to care; 3) resources available identified through social connections mapping; 4) trust in social connections; 5) community health promotion, prevention and management of NCDs. These are described below.

### Social risk factors and barriers to care

Social violence due to the long war and unrest suffered in the country, along with the on-going disputes among criminal gangs, has strongly affected communities’ health and their ability to secure continuity of care. This social violence has a long history, including a period of military dictatorship (1930–1979), the civil war (1980–1992) and the social consequences of a neoliberal economic and social policy (1992–2008). This protracted situation of social unrest had an important impact on the mental health status of the Salvadorian population, heightening the inability to manage chronic conditions. Many chronic patients interviewed related their illnesses to stress situations and shocks derived from this situation. More than a real causal relationship, it illustrates the tensions for the poorer layers of the population during that neoliberal period.

*They found I had high sugar, but at the beginning, the doctor told me that maybe it was just because of stress, because look, so much that we lived during the war, for 12 years. That is why one has acquired so much harm. Then, I went to consultation and the family doctor told me that these are the consequences of the war.* (Patient with DMT2).

During the interviews in primary health settings (phase 3), social violence, unhealthy lifestyles and economic barriers were mentioned as the main social risk factors for developing an NCD.

Health providers further identified lifestyle factors such as unhealthy diets as a major cause of presenting a chronic condition, highlighting that participant backgrounds and their economic situation need to be considered in order to facilitate access to healthy diets.

*The biggest challenge is that patients need to comply with taking their medication or following other recommendations. Especially when these recommendations have to do with lifestyle, principally with the diet.* (General practitioner).

*The economy is the main reason for not changing the diet, they look for the most economical way for their families and here the “pupusas”* [toasts made of corn and stuffed with beans, cheese and pork rinds], *are very common, all the people here eat them because they are cheap and accessible, most people eat fried meals even if it is so unhealthy.* (Health educator).

Patients also emphasized the high costs of healthy ingredients and water.

*It is expensive to maintain a healthy diet. I can’t, and not everyone has everything, we can only buy very little, and the bottled water.* (Man with hypertension and CKD).

In PHC settings, we observed a comprehensive approach to lifestyle behaviours by promoting community-based NCD management that considers the population’s social and economic environment. An example of this, as reported by one of the nurses of Chalatenanago, was a weekly local radio programme, set up by collaboration between the PHC centre of La Palma and the local school, which engages with adolescents to promote healthy eating and other healthy lifestyle behaviours.

### Patient pathways to NCD care

Given the above challenges, it was important to consider how these issues affect patient and provider experiences of NCD care. A chronic patient follows a clearly reported pathway. In the case of hypertension and DMT2, patients go to the PHC facility with the onset of symptoms such as fatigue. In some instances, patients may present at the PHC facility with chest pains or depression caused by the anxieties they feel given the on-going social violence.

*For me this started because of some problems that the family had with people. The problem was, they told me that they were going to kill a son of mine ... From that I was left with that distress. And from there I got a lot of affliction, I had a lot of sadness with a desire to cry, I had no joy, and a strong pain in my chest.* (Patient with hypertension).

For CKD patients, underdiagnoses are a major problem [25], with many only discovering their diagnosis when presenting a severe condition of their disease at the hospital. Therefore, the hospital (specialised care) is the entry point in many cases.

*Several factors may influence [why] they are seen at the hospital level, because they have not been followed previously in the first level of care and they go to the hospital when they are already serious, others because of lack of access.* (Intermunicipal coordinator).

In addition, CKD patients with a higher socio-economic status reported that they were diagnosed in private facilities, as there is still the belief that paying for medicines means they are better. Patients may also receive diagnosis during or because of an emergency, which often happens when public facilities are closed or there are long distances to get to these facilities.

*When I have an emergency, I prefer to go to the private clinic of San Salvador because there are no medicines in La Palma* [public PHC centre] *that can be given as good as in the private one. In the private clinic I do not wait, they attend you at the time you arrive.* (Patient with hypertension).

Health promoters play a very important role in identifying patients at risk of a chronic condition, showing that the promotion of community health is an important aspect of strengthening this health system.

*Our strategy is based on the fact that health promoters are the eyes of the health centre, of everything that happens in the community, they are the first to tell us that there is a person who might suffer from a chronic disease, knowing that, we schedule a visit to go to that patient’s home.* (Nurse).

Once patients are diagnosed, the follow-up is normally done in the PHC facilities, where they receive their medication and follow-up examinations. For this reason, the health reform reinforced the visits of specialised teams to the communities to assure a comprehensive approach for all, including remote areas, giving access to a gynaecologist, paediatrician, psychologist, physiotherapist, nutritionist, health educator, sanitary inspector and family doctor.

*The specialist physicians have programmed approaches to go to the communities, they evaluate the patients and identify those who need to attend a health facility or they leave them in control; in this approach, home visits can also occur and also there is a comprehensive family care visit at their home* (Departmental coordinator).

The referral system still lacks strong coordination within the different levels of care (specialised and primary care), but has been reported to have improved with the health reform [14, 15], giving patients a more comprehensive response to managing their disease.

*As family doctors, when a patient can no longer be given their medication or a disease is difficult to control, or if there is an emergency, we refer the patient to the hospital. For example, if it is a renal patient, there is a whole area for that, there they are assessed by an internist, and if [the internist] considers that the patient has to continue their follow-up there, the patient stays there. If not, they send us a referral to continue the patient’s follow-up here in PHC, engaging the patient in preventive activities with, for example, the health educator.* (Family doctor).

### Available resources

**Fig. 2:**
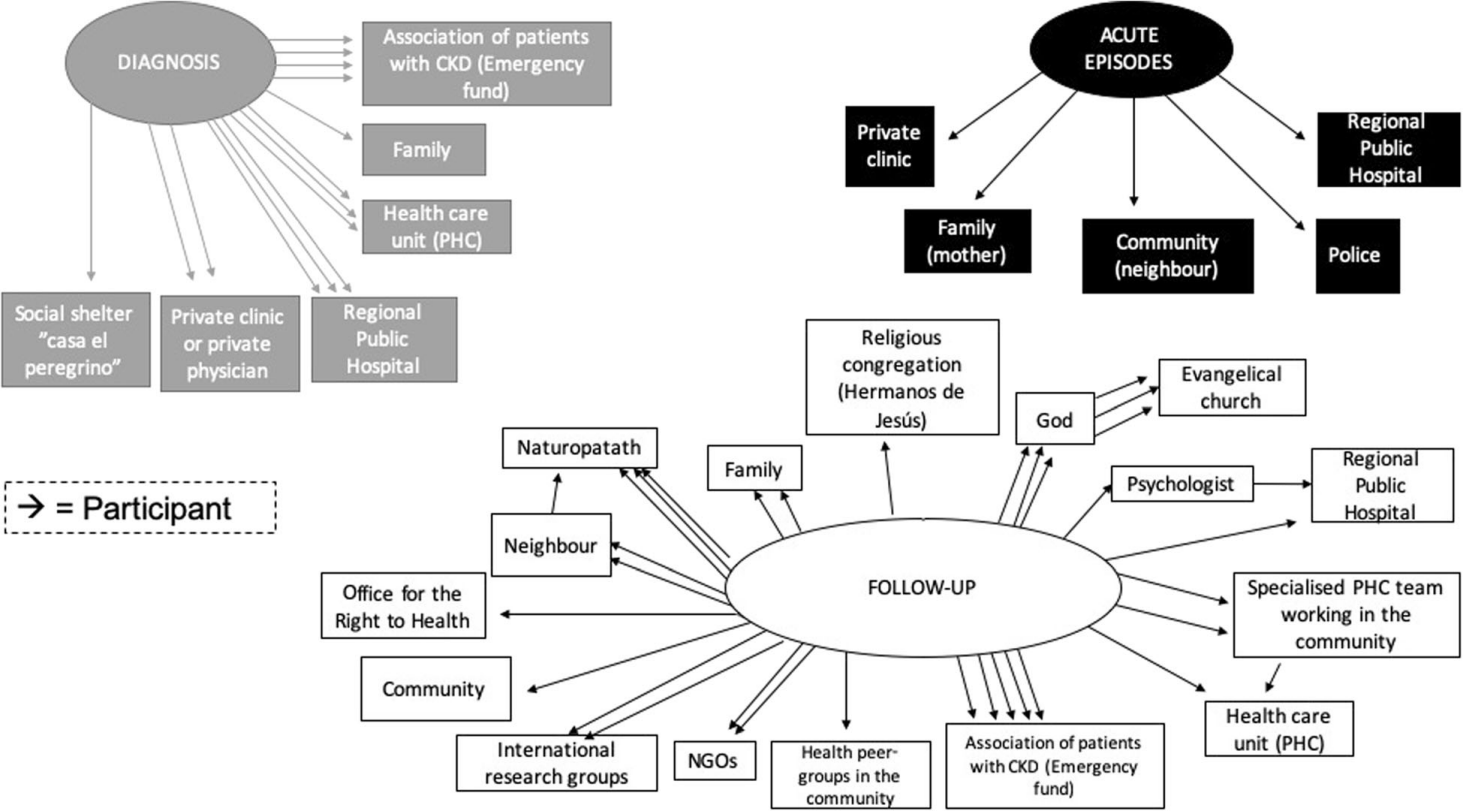
Workshop map of organised rural community

**Fig. 3:**
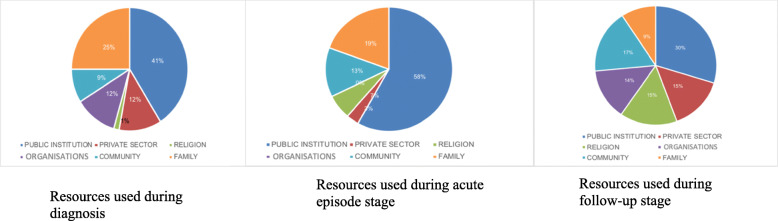
Resources used during diagnosis, acute episodes and follow-up stages

The social connections mapping exercise highlighted the resources available to, and used by, participants through the three stages of their illness: diagnosis, acute episodes and follow-up. Five workshops were held: three in rural settings and two in urban settings. Within each setting, a workshop was conducted in an “organised area”. These were identified and selected by a key stakeholder, the MoH. Organised areas were defined as those with higher social capital levels, social cohesion, and more presence of social and community organisations such as the NHF. Figures 2 and 3, respectively, show the identification and mapping of resources during a workshop and the resource used for each of the three scenarios: diagnosis, acute episodes and follow-up.

The hospital, as a public institution, is usually the place where people are first diagnosed. Additionally, public health campaigns account for high burdens of diagnoses, as many patients reported being diagnosed at their work place during annual examinations.

The same pattern is followed during acute episodes of the disease, with participants mainly seeking help from the private sector. This was reported as a change in patient behaviour, with the private sector used in cases of emergency.

*At the* [public] *clinic, normal exams are conducted and they do all of that, but they don’t give us what we need most, and this is specifically to do with our individual illnesses. For me, it’s my kidney and all my organs, and they can’t test for this in the clinic. I think a lot of people are dying because of this, because they can’t pay for this* [private clinic]. (Man with DMT2).

Following the health reform, access to remote communities has improved due to the implementation of specialised community teams [22]. However, emergencies often occur at times of difficult access to hospitals, for example, at night when there is limited public transport. This may lead to significant indirect costs, such as family members often having to be called upon to assist with financial support and to accompany the patient to the hospital.

The greatest variety of resources is used during the follow-up phase, with PHC playing an important role. Since the health reform, the public system, including extended first line services and the presence of supporting specialists, has reached more remote areas, and patients can ensure a follow-up of their illness in a comprehensive manner. Natural medicine is also widely used, since it is cheap and easy to access and there is a perception that there is no risk in its use. Extensive popular knowledge of these alternative medicines is in evidence.

Overall, in rural communities, private practitioners (mainly naturopaths) were regularly drawn upon during diagnosis and follow-up, due to relative ease of access and more economic options compared to urban areas. Religion is also predominant as an ‘institution’, as gathering in the church was seen to strengthen community cohesion, becoming more predominant as the disease progresses. A greater use of religious resources occurs during follow-up, revealing the relevance religion has in the management of chronic diseases, particularly for psychosocial aspects.

The most predominant organisational resource was the NHF, which played a predominant role in tackling social and health inequalities affecting chronic conditions such as access to clean water [25]. Finally, family support is evident throughout the progression of the disease and mainly used for transportation and social support.

### Trust in social connections

**Fig. 4:**
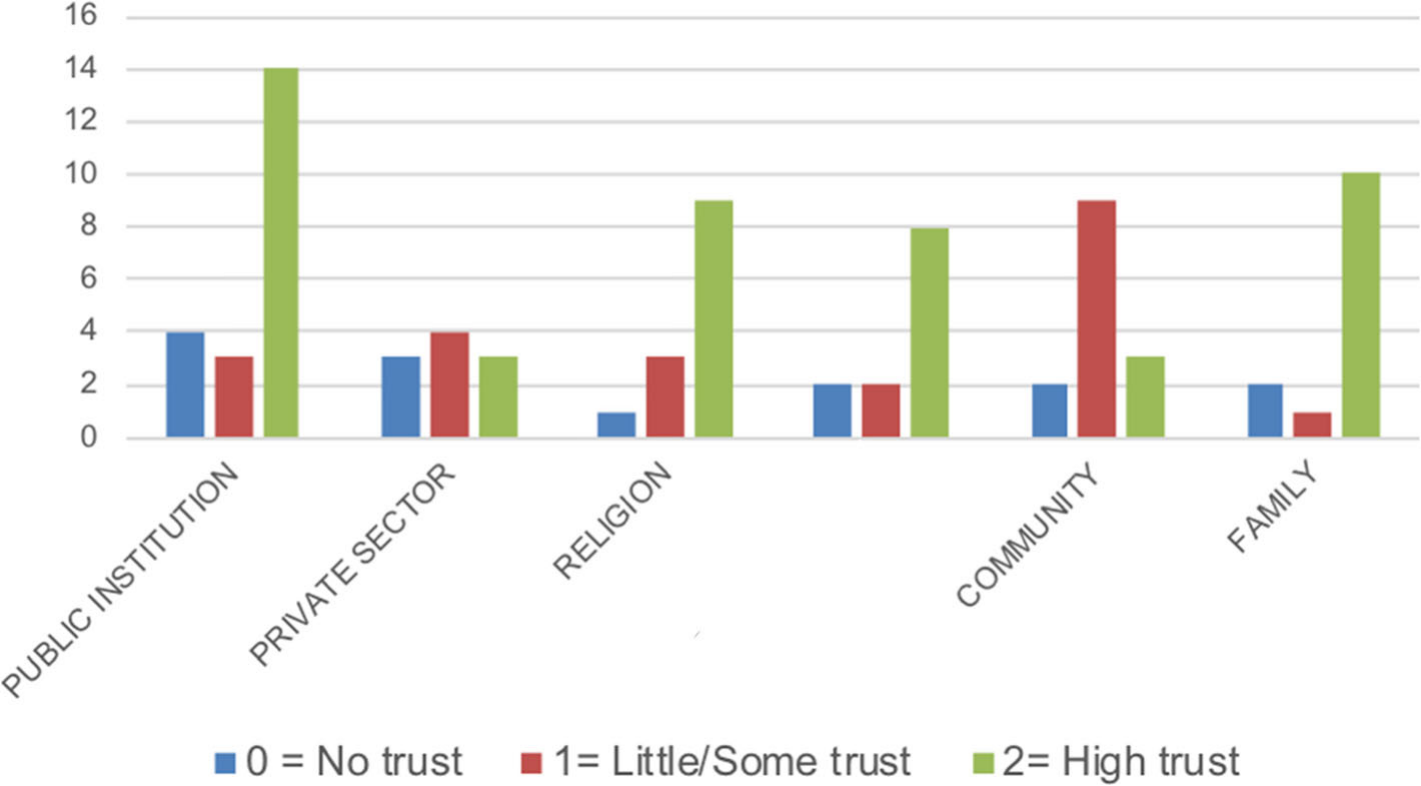
Levels of trust in resources used

As shown in Fig. 4, there were higher levels of trust in public institutions. This may have been.

due to greater trust felt in first levels of care (PHC), relative to trust placed in hospital care. In addition, access to free medicine following the abolition of fees for both medicines and consultation due to the health reform, may have also constituted a trust factor.

There were varied levels of trust expressed in the private sector. Participants had more confidence in examinations done in private rather than public centres, but there was less trust during follow-up due to economic constraints.

Regarding religion, the participants commonly referenced confidence in God and felt uplifted by the church environment.

*I think God gives us help and strength, because a lot of people I know with these illnesses have gotten very depressed and even suicidal. That’s why I think that being in a group and being in church helps to lift one’s spirits. Because God says that he is here.* (Woman with DMT2).

In our study, we included and compared communities with a strong level of organisation (in terms of social capital and social cohesion) with communities that had not yet organised themselves. Participants who belonged to an organised community felt empowered to form local associations. Although overall, there was not much trust in other members of the community due to the current situation of social violence. However, participants from organised communities reported high levels of trust in their communities and believed they could better respond to their health needs.

*Thankfully we are part of an organised community, because of this we formed an association. And in that association, we were able to get professional support. So, we had to form our own groups in order to mutually help each other.* (Woman with hypertension).

Gender differences were also observed while analysing levels of trust at the family level, with some positing higher levels of trust to female over male family members.

*...with men, it’s like they see the worst, they don’t place the same importance on things as women. We [women] are more careful to notice other people’s problems.* (Woman with DM).

### Community health promotion, prevention and management of NCDs

An interesting aspect of our analysis in El Salvador was the high level of organisation at the community level. It was reported by staff members that people had gained strong organisation levels during the civil war period and therefore there was a strong commitment to participate in decision-making. The NHF was born from people of the communities who commenced advocating for the right to healthcare in the early 90s. These community bonds were reinforced due to the need for organised activism for structural change to overcome the current social, economic and political aspects that impact on health. The NHF is present in 12 of 14 departments of the country.

Their perspective grants activist advocacy for comprehensive health policies, in which the structural causes of poverty and inequality are addressed through a whole society approach, linked to an extending network of empowering community activism and organisation.

*For us, there is strength in counting on social participation, because they* [NHF] *are in a way, the guards of the health system … Who better than they to visualise and follow-up, since a lot of the time we are not able to due to work … It is the communities that are organised, or those which the NHF has organised, that participate the most in our programmes, such as vaccinations, or controlling dengue fever and chronic illnesses. In the territories where the NHF is present, the population is very organised. The people know what their obligations are. We collaborate also, but they know that this is a shared responsibility … [empowered communities] know that health is for everyone, it is not just the responsibility of the MoH. It is not just about being cured, but it should be more about prevention. This is where the people of the NHF are helping the most.* (Regional Director of PHC centre).

In line with the *“health in all policies”* WHO framework, the community collaborates with the health system by identifying their needs and by participating in the design of strategies to improve NCD care and follow-up at community level. An example of this is in the region of Bajo Lempa where CKD is managed by involving all community assets. More attention is given to the prevention and early diagnosis of renal insufficiency linked to pesticide intoxication, known as chronic interstitial nephritis of agricultural communities (CINAC). In this region, the promotion of community peer support groups for NCDs where patients can learn from each other is advocated, as further described in another study conducted by the same research team [25].

## Discussion

In view of the growing burden of NCDs worldwide, the experience in El Salvador on the development of a comprehensive strategy of NCD management highlights the following findings:

The combined use of qualitative data collection methods within different levels of care and at the community level help to better understand the comprehensive approach that is needed in NCD management. By investigating the experiences of health care staff and people living with an NCD in El Salvador, social risk factors and barriers to care were identified as having a strong impact in the management of NCDs. Lifestyle factors and economic barriers were also mentioned as social risk factors affecting the ability to maintain the type of healthy lifestyle behaviours needed for preventing and presenting with NCDs.

Regarding patient pathways to care, the implementation of PHC has enabled a more accessible NCD follow-up with a holistic approach by combining a multidisciplinary PHC staff with specialised teams going to communities.

A clear change in health seeking behaviours was noted when assessing the available health resources. Patient diagnosis, still highly dependent on the private sector up until follow-up, showed that psychosocial needs were mainly met through family support and religion. Further, organisations such as the NHF are used to empower patients. This has improved shared decision-making, leading to a better understanding of patient health needs.

Participants from organised communities also reported high levels of trust in their own communities and believed they were more responsive to their health needs.

Finally, concerning community health promotion and prevention of NCDs, highly organised communities are participating in the identification of the needs and in the design of strategies to improve NCD care and follow-up at community level. Social health movements, such as the NHF are promoting policies and strategies, which consider the population needs.

The findings highlighted that community-based NCD programmes such as those included in this study could potentially be useful in other settings. The essential elements for adapting these to other contexts relate to the role of the organised community as a complementary approach for supporting health services [10, 26]. Elsewhere it is suggested that community-based interventions are useful for supporting individuals, families and communities to overcome obstacles, such as ensuring continuity of care from diagnosis to follow-up, which is achieved through the guidance of the health services and well-developed NCD guidelines [27].

A study reporting recommendations for the management of NCDs in conflict areas [28] highlights that an integrated, multi- and interdisciplinary approach should be developed, ensuring the provision of quality care involving community leaders and influential individuals. Such an approach, as reported by Magnusson et al. [29] needs to be grounded in the right to health. This includes the prevention of NCDs and their risk factors, the improvement of access to care, and addressing the social impact of these ailments. This highlights the appropriateness of a strategy under the *health in all policies framework* [30] as carried out by the NHF.

Over the last 10 years (2009–2019), El Salvador has implemented a national health system with the aim to guarantee universal health coverage. Progress has been made, but the fragile context remains challenging, not only to ensure further progress, but also to maintain what has been accomplished [31].

The Latin American current of critical epidemiology [32] criticises the concept of social determinants of health on the basis of its fragmented analysis of the social reality. They instead advance the concept of *social determinationof health,* as this approach emphasises the determining influence of the overarching structural inequalities and the skewed power relations at all levels, which help us to better understand the complex interactions of these layers on the social and health reality of communities and individuals.

This public health trend explicitly links their analysis to organised activism for structural change [33], advocating for an extending network of empowering community activism and organisation [34, 35]. Consequently, health promotion then refers to a process where individuals organise and act to increase control over factors related to their individual and collective health [36, 37].

While this approach is widely embraced in communities that engage with the NHF, this type of organised engagement has a clearly political stand, implying a continuous struggle to maintain their influence on local health policies through social mobilisation. While over time the number of organised communities and their impact has been increasing, their focus is mainly on rural areas, rather than cities. Moreover, other social phenomena cause contrasting dynamics: youth gangs create high levels of social violence, related to the enormous economic divide in the country.

Political accountability mechanisms ensured through the NHF constituted an important factor to rate higher trust in health system performance. Accordingly, our study found that quality of the management of NCD care lies in a comprehensive approach to improve the relation between PHC and specialised environments, relying on community-based interventions involving all community assets and taking into account the social and economic context.

### Limitations

The complexity of the sociocultural, political and economic context of El Salvador must be noted. The periods of fieldwork may have been too short to gain an overall in-depth knowledge of this context. Moreover, the high level of violence in important parts of the country limited the fieldwork possibilities. Many of these challenges were partially solved thanks to the support and accompaniment of key Salvadorian stakeholders.

This study was conducted between November 2017 and January 2019. The results of the national elections in February 2019 led to a government change, which might imply important changes in the health system analysed here.

## Conclusion

The complementary research methods and the different groups of participants (patients, health personnel and health system coordinators) all identified similar strengths and obstacles in NCD management. The combined use of these methods proved effective for obtaining an in-depth understanding of NCD management strategies in El Salvador along the different levels of care (PHC and specialised care), and could serve to guide analyses in function of policy-making in other fragile contexts.

The generation of equitable health policies that pursue accessible quality services is an essential first step to improve access and continuity of care for NCDs. However, the Salvadorian experience also shows that a strong and well-organised involvement of the communities can positively influence health care access at the community level, while also promoting a prevention culture.

Participants insisted on the strengthening of healthy lifestyle initiatives adapted to their sociocultural and economic contexts.

## Supplementary information

**Additional file 1.** Interview guides; File containing interview guides used during phase 1 and 3 of data collection.

## Data Availability

The datasets used and analysed during the current study are available from the corresponding author on reasonable request.

## Abbreviations

CINAC: Chronic interstitial nephritis of agricultural communities
CKD: Chronic kidney disease
DMT2: Diabetes mellitus type 2
FMLN: Farabundo Marti National Liberation Front
LMIC: Low- and middle-income countries
MoH: Ministry of Health
NDC: Non-communicable disease
NHF: National Health Forum
PHC: Primary health care
QMU: Queen Margaret University
RUHF: Research Unit on Health in Fragility
UN: United Nations
WHO: World Health Organisation
