# Supplementary material for: Community and health staff perceptions on non-communicable disease management in El Salvador’s health system: a qualitative study
Source: BMC Health Serv Res. 2020 May 27;20:474. doi: 10.1186/s12913-020-05249-8 (PMC7251854; doi:10.1186/s12913-020-05249-8)
Supplement: Supplementary file 1 — Additional file 1. Interview guides; File containing interview guides used during phase 1 and 3 of data collection. [file 12913_2020_5249_MOESM1_ESM.docx]

***Patient interview guide: in-depth interviews for illness narratives (phase 1)***

Interview identifier: ____________ Date: ___/___ __/__ __

Interviewer: ____________________________________________

Note taker: _____________________________________________

Patient details:

Sex: Male: ___ Female: ___

**Welcome and introduction**

- Introduce the aims and objectives of the study and the expected role of the participant (i.e. today we will be asking you questions about your life and experiences concerning your health. We are interested in finding out about your health history, they types of support you are familiar with and/or have received, any changes you have experienced while seeking health care, any expenditures you have incurred and your overall wellbeing)
- Explain why this participant has been selected for interview
- Review the information sheet along with the participant and ensure questions are answered
- Ensure confidentiality, anonymity and participant’s right to decline to answer or leave interview at any time
- Remind participant of interview duration (2-3 hours)
- Signing of consent form

**1. Participant background (sociodemographic characteristics)**

Family structure:

- Do you know your age? If so, how old are you?
- Do you know where you were born? If so, where?
- How many people were in your home when you were growing up? Did you have brothers or sisters? If so, how many? Tell me about your extended family.
- Did you go to school? If so, for how long/up to what grade?
- Are you married? If so, does your spouse live with you in your home? If not, have you been married before?
- Do you have children? If so, do they live with you in your home?
- How many people currently live with you in your home? How many women? How many men? Children? Grandchildren? Does everyone sleep under the same roof? Eat in the same kitchen?

Household economy

- Who is the main financial provider in your household. In which sector? (e.g. agriculture, industry) How long has this person been in this job? How often do they get paid?
- What do you do to make money?
- Is there anybody else in your household that earns money? If so, what do they do?
- Do you or anyone in your household receive any form of financial help? (e.g. subsidies)
- Please tell me about your household. (e.g. how much land? How many animals)
- From where do you obtain water? (e.g. does your house have running water or do you have to source your water elsewhere such as the community or a well)

**2. Important life events (focusing on health history)**

- We would like to visualise your life and your experiences. Can you please draw a line (provide paper) that represents your life? On the line, please mark the events that you consider most important including when they occurred.
- We would also like to understand your health history. On the line, can you please mark any important episodes you can recall concerning any of the conditions you have or have had (e.g. diagnosis, need for emergency care)

Health episodes

- Do you currently have a chronic illness or condition? (e.g. hypertension, cardiovascular disease, chronic kidney disease, diabetes)
- For each mentioned either now or in the past:
  - Please describe the illness/episode.
  - What happened?
  - How did this happen?
  - When and how long did the episode last?
  - What other problems did you experience that may have been related to this illness/episode? (health or otherwise)
  - Have you been provided with information concerning your illness? Who provided this information and/or where did you access it?)

Health service (e.g. type of service: public or private, level of service)

- For each of the above mentioned:
- What type of health service did you use?
- Why did you choose this service?
- How did you decide where to go?
- What information did they give you about your illness?

Care received

- Please tell us about your experiences with the different types of care services you have used (e.g. quality of care, cleanliness of facility, privacy)
- How long did you have to wait to receive care (e.g. whether within the facility or for referrals)?
- How do you feel you were treated during your visits?

Cost of care

- Did you have to pay any fees for your visit?
- How much was this?
- How did you pay for this?
- Were you able to cover the costs yourself?
- Did you receive any help?
- What did you do if you weren’t able to pay?
- Did this affect your ability to pay for your other needs in any way? If so, how?
- Did you incur any other costs due to your visit? (e.g. transport, medication, out of pocket fees)
- Is there anything that you weren’t able to pay for? If so, what?
- Does your illness affect your ability to make money? If so, how?

Medication

- Are you currently taking any medication for your illness?
- Was this prescribed or did you purchase this over the counter?
- Where did you obtain your medication?
- What was the cost and were you able to pay for this? If not, what was the reason for this? (e.g. head of household unable or unwilling to pay)
- Did you receive any help paying for this?
- Have you ever had to change your treatment regimen? If so, what were the reasons for this? (e.g. medication too expensive, unavailable or out of stock)

Family illness

- Has anyone in your family or household experienced a significant health episode?
- Please tell us about any events that may have changed your household structure? (e.g. changes to numbers of men, women, children)
- Have there been any deaths in your family or household?
- Has anyone moved away? If so, was this a temporary or permanent move?
- When did this happen?

**3. Changes in perceptions of care over time**

- How have you felt with the quality of care you received? Has this been different in different moments in time?
- Have you felt that the quality of care you received has gotten better, worse or has been without any noticeable changes over time? How so?
- (If changes have been noticed) Why do you think these changes occurred?
- Have there been any changes in costs of healthcare over time? Please explain.
- Why do you think this has happened?
- Has this affected the way you have used services?

**4. Policies to aid accessibility of services**

- Are you familiar with any policies that are in place to help people access and pay for health services? (e.g. free healthcare for under 5s). If so, do you know anyone who has benefitted from these policies?
- Are you familiar with any policies to help people with chronic illnesses? If so, do you know anyone who has benefitted from these policies?
- Do you feel that these policies work/don’t work? Why or why not?
- Do you feel these policies are equally accessible for everyone?
- What do you think would be most beneficial to help you and your family to access health care?
- Do you have any recommendations?
- Do you have any questions for us?

**End of interview.**

***Patient interview guide: semi-structured interviews for understanding pathways to care (phase 3)***

Interview identifier: ____________ Date: ___/___ __/__ __

Interviewer: ____________________________________________

Note taker: _____________________________________________

Patient details:

Sex: Male: ___ Female: ___

**Welcome and introduction**

- Introduce the aims and objectives of the study and the expected role of the participant (i.e. today we will be asking you questions about your life and experiences concerning your health. We are interested in finding out about your health history, they types of support you are familiar with and/or have received, any changes you have experienced while seeking health care, any expenditures you have incurred and your overall wellbeing)
- Explain why this participant has been selected for interview
- Review the information sheet along with the participant and ensure questions are answered
- Ensure confidentiality, anonymity and participant’s right to decline to answer or leave interview at any time
- Remind participant of interview duration (1 hours)
- Signing of consent form

**1. Participant background**

- Do you know your age? If so, how old are you?
- Do you know where you were born? If so, where?
- How many people were in your home when you were growing up? Did you have brothers or sisters? If so, how many? Tell me about your extended family.
- Did you go to school? If so, for how long/up to what grade?
- Are you married? If so, does your spouse live with you in your home? If not, have you been married before?
- Do you have children? If so, do they live with you in your home?
- How many people currently live with you in your home? How many women? How many men? Children? Grandchildren? Does everyone sleep under the same roof? Eat in the same kitchen?
- Please tell me about your household. (e.g. how much land? How many animals)
- From where do you obtain water? (e.g. does your house have running water or do you have to source your water elsewhere such as the community or a well)

**2. Pathways to care**

- Do you have a chronic illness or illnesses? If so, tell me about it.
- When was the first time you sought and/or received care for your symptoms?
- What happened next? Where and when were you diagnosed?
- Was anybody with you when you were diagnosed? Did you tell anybody about your diagnosis?
- When did you begin to receive care in this facility? (probe: accessibility)
- Is there a particular day/time that you prefer visiting the facility for your treatment?
- How long does it take you to reach the health facility?
- What kinds of costs do you incur to reach the facility and/or during your visit? (e.g. transport, fees)
- Please tell me about any challenges you experience managing and treating your illness.

**3. Health episodes**

- Have you ever had any complications or important health episodes since you have been diagnosed?
- For each episode:
  - What happened?
  - How did this happen?
  - When and how long did the episode last?
  - What other problems did you experience that may have been related to this illness/episode? (health or otherwise)
  - What did you do? Did you visit any health services? Where did you go to receive care?
  - How did you decide which facility/health service to use

**4. Quality of care**

- During your visits to the health facility:
  - Do you feel you receive sufficient attention from the health care staff?
  - How long do you generally wait before you are seen?
  - How many staff members are you in contact with during your visit? For how long with each one?
  - Do you feel you are given sufficient time with each staff member you are in contact with?
  - Do you feel you are given enough information about your illness during your visits? Is there anything you feel is lacking?
  - Do you feel you receive enough support for your treatment? Is there anything you feel is lacking? Is there anything you feel occurs exceptionally well? Please explain. (probe: communication, technical medical language)
  - Have you been in any situations where it was difficult to access or reach the health facility? (e.g. illness, transport, violence)
  - How did you resolve this? Did any of the health service staff assist you? If so, in what ways?
  - How do you feel the other patients are treated by the health care staff? How about you?

**5. Help and support**

- Do you feel the health care staff support you in following your treatment? Is there anything you would like to see change?
- Who provides you with the most support during your treatment? (e.g. health care staff, family members). How so?
- Do you take part in any support groups or are you a member of any organisations in your community? If so, please tell me about it. What type of support does this provide?
- Are there any support groups specific to your illness?
- What kind of financial support is available to your household? Who provides the majority of the financial support? From which sector do you and other members of your household gain support? How often do you receive this financial support (e.g. hourly/daily/weekly/monthly salary)
- Do you receive any government subsidies?

**6. Changes in perceptions of care over time**

- Can you explain what kinds of changes have occurred over the past 10 years? Have these affected the way you have been able to manage your illness? If so/not, please explain.
- Do you feel the situation has gotten better or worse? (i.e. either situation social violence, personal circumstances, or ability to manage illness)
- (If changes have been noticed) Why do you think these changes have occurred?
- Have health care costs changed over time for you? Please explain.
- Have changes to health care costs affected your ability to seek or receive care? Please explain.
- Do you have any suggestions as to ways you would like to see primary health care services change or improve?
- Do you have any questions for us?

**End of interview.**

***Staff interview guide: semi-structured interviews for understanding pathways of care (phase 3)***

Interview identifier: ____________ Date: ___/___ __/__ __

Interviewer: ____________________________________________

Note taker: _____________________________________________

Employment title:

**Welcome and introduction**

- Introduce the aims and objectives of the study and the expected role of the participant (i.e. today we will be asking you questions about your work and your experiences providing health care for NCD patients)
- Explain why this participant has been selected for interview
- Review the information sheet along with the participant and ensure questions are answered
- Ensure confidentiality, anonymity and participant’s right to decline to answer or leave interview at any time
- Remind participant of interview duration (1 hours)
- Signing of consent form

**1. Profile of participant and role in PHC**

- Please tell me about your work and what you do (e.g. background, training, history of involvement with PHC and/or services)
- (If not already mentioned above) What is your role in providing PHC and/or services for NCD patients***?***

**2. Understanding patient trajectories at the first level of care**

- Please describe your involvement with NCD patients in terms of the communication and activities you fulfil at different stages of their care-seeking process (e.g. at reception, patient registration, risk assessment, follow-up clinical, routine patient visits)
- What are the main challenges you face in fulfilling your role (e.g. when it comes to screening, diagnosis, treatment follow-up, patient social and environmental conditions)?
- What strategies do you use to respond to these challenges (probe on patient follow-up methods, frequency of follow-up attempts, referral to other staff or external support services)

**3. Role in NCD care**

- (For all health and/or social service staff) Please describe the steps you take when attending to patients with chronic illnesses.
- (For community care staff) Please describe the steps you take to locate or reach NCD patients.
- Please tell me about your experiences providing support to NCD patients. Can you provide any recent examples of some of the work you do with patients. Were there any problems or challenges you had to deal with? Please explain.
- How feasible is it to provide more support to patients who need this? (e.g. practicalities reaching patients, sustainability)
- What are some of the challenges you face in providing support to NCD patients? (communication barriers, literacy, social and/or economic barriers) Do any of these occur more frequently than others? Why do you think this is?
- Have you noticed any differences or changes in your interactions with NCD patients since the health reform was implemented? (e.g. frequency of patient visits, patient attitudes)

**4. Strategies for managing NCD patients**

- Are there any other challenges or problems that you encounter when interacting with NCD patients? How do you manage these?
- Are there any strategies that you use or that you have seen other people use when it comes to facilitating communication with NCD patients? (i.e. in order to ensure patient comprehension of services and care)
- Please describe any experiences you may have had, if any, with patients who you feel were more receptive to the information you were providing them. How about patients who expressed disinterest/disengagement in their treatment or with the medical advice they are given Describe how you handled this.
- Do you have any recommendations for improving communication with NCD patients? (e.g. in order to ensure they are able to manage their illness)

**5. Experiences providing care following the health reform**

- Have you noticed any changes in the way care is provided to NCD patients since the implementation of the health reform?
- Have you been able to strengthen your capacities in any way as a result of the health reform?
- Tell me about your experiences with the mechanisms that have been put in place to ensure early diagnosis.
- Tell me about your experiences with the mechanisms that have been put in place to ensure medical supplies are sufficient.
- What sorts of activities are in place to ensure early detection and/or risk factor screening?
- Please tell me about the different prevention, promotion, care and follow-up activities that you deal with in your facility.
- What have you found most challenging since the health reform? Least challenging? (e.g. time needed for paperwork, practical challenges with implementation)
- Do you have any questions for us?

**End of interview.**
